# Comparison of Eyemetrics and Orbscan automated method to determine horizontal corneal diameter

**DOI:** 10.4103/0301-4738.62647

**Published:** 2010

**Authors:** Arvind Venkataraman, Sapna K Mardi, Sarita Pillai

**Affiliations:** Vasan Eye Care Hospital, Chennai, India

**Keywords:** Corneal diameter, Eyemetrics, Orbscan, white to white

## Abstract

**Purpose:**

To compare horizontal corneal diameter measurements using the Orbscan Eyemetrics function and Orbscan corneal topographer.

**Materials and Methods:**

Seventy-three eyes of 37 patients were included in the study. In all cases, the automated white-to-white (WTW) measurements were obtained using Orbscan by two observers. Using the Eyemetrics function, the WTW was measured manually by the same observers from limbus to limbus using the digital caliper passing through the five point corneal reflections on the Orbscan real image. The data was analyzed using SPSS software for correlation, reliability and inter-rater repeatability.

**Results:**

The mean horizontal corneal diameter was 11.74 ± 0.32mm (SD) with the Orbscan and 11.92 ± 0.33mm (SD) with Eyemetrics Software-based measurement. A good positive correlation (Spearman r = 0.720, *P* = 0.026) was found between these two measurements. The coefficient of inter-rater repeatability was 0.89 for the Orbscan and 0.94 for the Eyemetrics software measurements on the anterior segment images. The Bland and Altman analysis showed large limits of agreement between Orbscan WTW and Eyemetrics WTW measurements. The intra-session repeatability scores for repeat measurements for the Orbscan WTW and Eyemetrics measurements were good.

**Conclusion:**

Eyemetrics can be used to measure WTW and the Eyemetrics measured WTW was longer than the WTW measured by Orbscan.

Horizontal corneal diameter has been used in the selection of intraocular lenses (IOLs) to be placed in the sulcus and anterior chamber.[1–7] With the usage of phakic intraocular lenses (pIOL) it has become a great challenge to accurately measure the white-to-white (WTW) since the selection of an appropriate sized lens would determine the effectiveness and long term stability of the lens. Specifically, with angle-supported pIOLs, selection of the proper haptic size is important. An oversized or undersized pIOL can induce unwanted effects such as lens decentration, pupil ovalization, excessive mechanical rubbing against anterior segment structures with resulting flare reaction and angle closure glaucoma.[6,8–11]

Some of the newer generations of IOL formulas, like Holladay 2, need the correct WTW length to determine the effective lens position in normal as well as post corneal refractive surgery patients.[12,13] According to the older literature, normal values for the WTW distance in adults is 10.60 mm for the vertical diameter and 11.70 mm for the horizontal diameter (range 10.5 to 12.75 mm). Probably these data are from manual measurements from an era when no automated systems were available.[14,15] WTW measurements are done using several methods such as with a millimeter ruler or scales in slit-lamp oculars. Recently, computer-assisted devices for comprehensive analysis of the anterior eye segment have been developed.[16,17] Of these, the one most commonly used is Orbscan (Bausch and Lomb) corneal topography. Though reliable, it can, very occasionally, give a wrong estimate of WTW, which can cause disastrous consequences, especially in posterior chamber pIOL implantation.[6]

The objective of this study was to compare the Orbscan (Bausch and Lomb) derived automated WTW measurement with that of Eyemetrics (Bausch and Lomb) based measurement of WTW on the Orbscan image and to demonstrate the differences and similarities of the two measurements.

## Materials and Methods

The study comprised of 73 eyes of 37 patients (25 females and 12 males). None had any previous ocular surgery or disease affecting the cornea or sclera. The Orbscan topographer used digital image processing for WTW measurements. The subject's head position was secured by chin and forehead rests, and the subject fixated on a light inside the device. The operator adjusted the distance between the eye and device by focusing on the test marks projected onto the cornea. Information about the corneal shape, corneal thickness and the anterior chamber depth was acquired by scanning the anterior eye segment with a slit beam. A digital grey scale image of the anterior segment was reconstructed from 140 slit images. The computer automatically detected the corneal limbus by comparing the gray scale steps and calculated the corneal diameter.

Using the Orbscan topographer, three consecutive automated measurements were taken by two different examiners for the right and left eye respectively and the mean for each eye was calculated. Two examiners independently measured the horizontal corneal diameter using the Eyemetrics function (digital caliper in Orbscan) twice consecutively.

**Figure 1:**
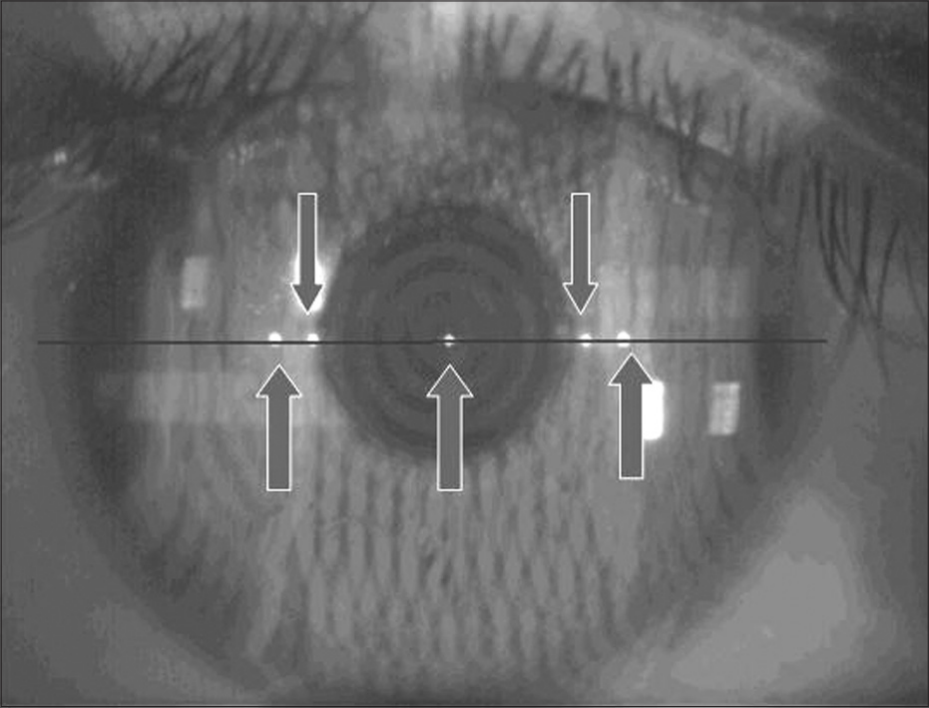
The real image of cornea with Eyemetrics digital caliper measurement along the 5 point corneal reflections

The WTW was measured in the following manner: The gray scale image was opened using the Eyemetrics function in the Orbscan. The brightness of the image was adjusted to clearly visualize the limbus. Then using the digital caliper, the limbus on the left side of the image was marked by the click of the mouse. A line was drawn along the five point-reflection seen on the real image to the opposite limbus [Fig. 1] and the measurement was automatically displayed on the screen.

The correlation between the two WTW measurements in right eyes and left eyes was analyzed, and the Spearman's correlation coefficient for each anatomical parameter obtained. As the correlation between eyes was not confirmed, an analysis of the correlation between the two WTW was performed in which each eye was considered an independent variable. Then, the Spearman's correlation coefficient was calculated and linear regression analysis performed.

The data was analysed for correlation, reliability and inter-rater repeatability. The interchangeability of the 2 measurement devices were analyzed using the Bland and Altman[18] method. The Bland and Altman plot shows the differences in the measurement of 1 specific parameter between the compared methods plotted against the average of the mean results obtained with both methods.[19]

## Results

**Figure 2:**
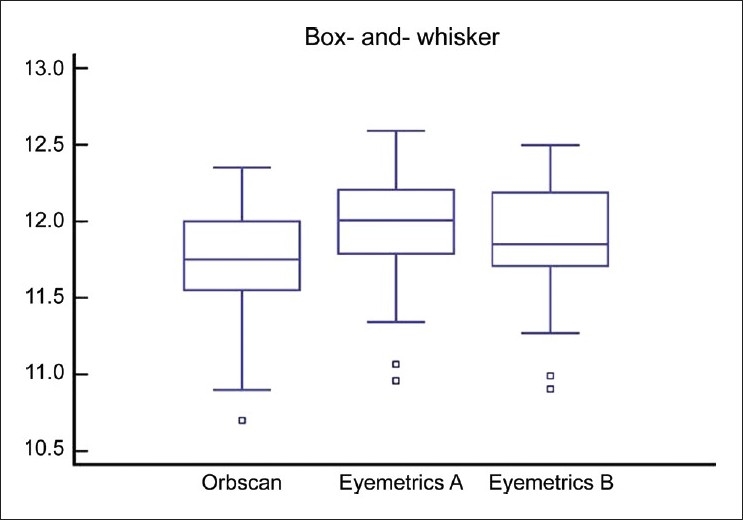
Box and whisker plot showing the distribution of measurements for Orbscan and Eyemetrics measurements for examiner A and examiner B

**Figure 3:**
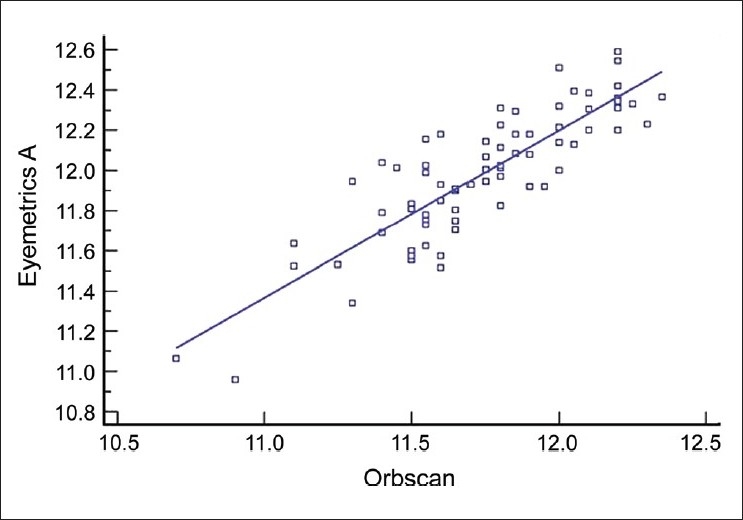
Scatter plot showing the relationship between Orbscan and examiner Bs Eyemetrics white-to-white measurements

**Figure 4:**
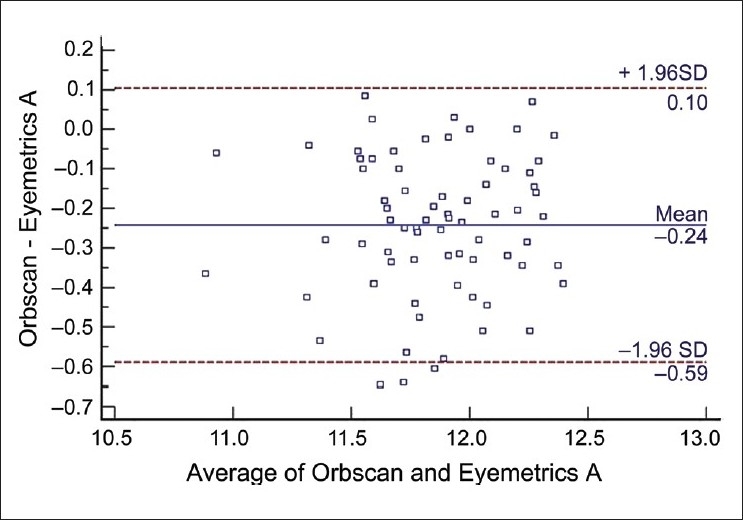
Bland-Altman plot. The differences between Orbscan and Eyemetrics (examiner A) white-to-white distances are plotted against the mean value of both. The upper and the lower lines represent the limits of agreement

**Figure 5:**
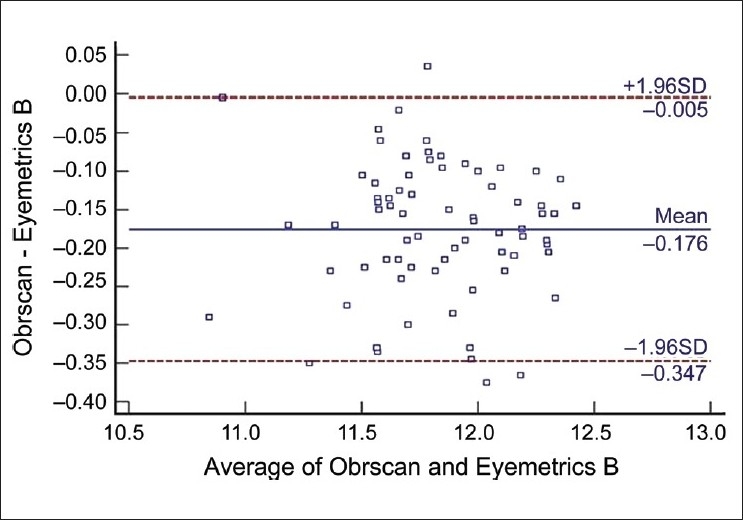
Bland-Altman plot. The differences between Orbscan and Eyemetrics (examiner B) white-to-white distances are plotted against the mean value of both. The upper and the lower lines represent the limits of agreement

The mean corneal WTW diameter was 11.737 ± 0.32 for examiner A and 11.739 ± 0.33 for examiner B with Orbscan automated measurement. The Eyemetrics average horizontal corneal diameter measurement was 11.99 ± 0.37 for examiner A and 11.92 ± 0.33 for examiner B [Fig. 2]. The mean values with the Orbscan and Eyemetrics showed no statistically significant difference between the two examiners. The difference in mean between the two measurements of WTW was −0.17 (range −0.37 to 0.03). The LoA (mean ± 2 standard deviations of the differences) were smaller in the Orbscan measurements than in the Eyemetrics measurements. Fig. 3 shows a scatter plot of the relationship between variables. There was a positive and statistically significant correlation between Orbscan WTW and Eyemetrics WTW distances (Spearman r = 0.720, *P* = 0.026). The linear regression analysis then revealed a reasonable model with a good predictability (R^2^ = 0.444). The coefficient of inter-rater repeatability was 0.89 for Orbscan measurement and 0.94 for the Eyemetrics measurements. The Bland Altman plots showed that the measurements were comparable [Figs. 4 and 5].

The difference between the two parameters was statistically significant (*P* = 0.005) (nested mixed effects ANOVA). No statistically significant interaction effects were detected between eyes or persons. The Orbscan automated measurements were statistically different from that of Eyemetrics based measurements for both the examiners A and B (*P* < 0.001). But there was no statistically significant difference in WTW measurements between the two examiners using Eyemetrics function.

## Discussion

Several studies have reported horizontal and vertical corneal diameter or WTW data in normal populations.[14,15] Different methods have been described for measuring these distances. These methods can be divided into two groups- manual and automated, depending on the examiner's level of participation in the procedure. Millimeter rules, calipers, gauges, or scales in slit-lamp oculars are devices for manual determination of the WTW diameter. The accuracy of limbus recognition by computer software of the automated methods depends on the quality of the anterior segment images. With the Orbscan topographer, this is composed of a series of slit lamp images. The IOL Master, however, measures the WTW based on a digital “photographic” image that it acquires. This instrument then digitally locates the limbus based on a sudden change in the contrast from bright sclera to dark cornea. This contrast difference can vary depending on illumination and quality of the image.

An incorrect sized angle-supported pIOL can induce complications such as decentration, inflammatory reactions, and glaucoma or pupil distortions due to the excessive pressure of the haptics over the angle structure.[20] If the phakic lens placed in the posterior chamber is smaller than that of the sulcus diameter it might touch on the lens causing cataract or can rotate in the sulcus which in the case of a toric lens can cause abnormal shift in refraction. Hence, analysis and evaluation of each case individually using appropriate devices is crucial.

Comparisons using data from different studies should be performed with caution to avoid wrong conclusions as the studies analyzed different cohorts of eyes.[21–23] The measurements done manually with a digital caliper, may be imprecise in some cases because of difficulties in accurately defining the end of the cornea and the beginning of the sclera especially when the image is not focused on the limbus but rather on the cornea.[24] This defocus contributes to the potential source of error in selecting the endpoint. But images obtained in our study were by using the Orbscan. As mentioned earlier, the Orbscan images composed of a series of slit lamp images are of better quality. Other studies have mentioned difficulty in the detection of the exact point where the sclera begins without using magnification, resulting in variability in measurements using manual calipers and rules.[25] In the Eyemetrics system the contrast of the picture can be adjusted so that the corneoscleral junction can be easily identified. We have also mentioned the five point-reflections on the image of the cornea. Measuring along these five point -reflections avoids measuring WTW eccentrically on the cornea thus reducing errors in measurement. In our study, we found excellent intrasession scores, as well as good inter-observer repeatability that confirmed the consistency of the data obtained with the Eyemetrics systems. We attribute this to the points mentioned earlier as advantages of the Eyemetrics system. Manual methods described in different studies differ significantly and have a relatively great range of variance.[25] The comparatively poor repeatability of the caliper may be because the eye must be touched with the caliper tips. Least count of a surgical caliper is 1 mm. This can cause involuntary defensive eye or head movements that make the measurements more difficult. In our study, the variability is less compared to other studies since the measurement was performed on the real image and the caliper used is the Eyemetrics software specifically developed to measure on the image captured.

A study by Baumeister *et al*. has shown that measurements of mean absolute WTW measurements with Orbscan were shorter by 0.24 mm compared to that of IOLMaster.[25,26] They have also found that the repeatability of measurements with Orbscan is not as good as IOLMaster. The study by Pinero *et al*. found that measuring WTW using digital calipers in CSO (Costruzione Strumenti Oftalmici) corneal topography system measures longer WTW than that of automated measurements.[24] These findings were similar to our finding that the Orbscan measured WTW was on average shorter by 0.19 mm than that of Eyemetrics measured WTW. But the WTW calculated by the IOLMaster cannot be used in choosing the correct Visian^®^ implantable collamer lens (ICL) since the software developed by the STAAR^®^ Surgical Company requires only Orbscan WTW.

In our study, we have seen that the Orbscan measured WTW is less than that measured by Eyemetrics measurement. This has implications, mainly in posterior chamber pIOL implantation, anterior chamber lens implantation, posterior chamber sulcus and scleral fixated lenses. Gimbel *et al*. reporting on one-year outcomes of toric pIOLs mentioned that they had a learning curve in selection of ICLs.[6] When they were using ICLs as advised by the software which is 0.2 mm greater than the Orbscan measured WTW they observed more rotational instability and decreased vaults. As a result they started using a posterior chamber pIOL not less than 0.3 mm longer than that of WTW even if it is one step longer than that suggested by the software.

Our study finds that WTW is underestimated by Orbscan when compared to that of Eyemetrics software derived WTW measurements. The probable reason might be that the automated measurement of color change at the limbus is not very sensitive to subtle variations that can happen in individual patients.

This study shows that Orbscan corneal topographer underestimated WTW measurements in comparison to the Eyemetrics measurements. This observation is similar to the studies of WTW measurements using IOL Master. This underestimation of WTW can be identified and corrected by a countercheck using Eyemetrics software. Further studies comparing Eyemetrics WTW and Orbscan WTW measurements in patients with pannus and other peripheral corneal changes are required. This method is definitely a useful supportive tool for automated measurement of corneal diameter in selection of pIOLs and ACIOLs.

## Conclusion

Automated Orbscan measurements underestimate the WTW length when compared to that of Eyemetrics measurements. Use of Eyemetrics, which is a part of the Orbscan, will increase the accuracy of WTW measurement and hence avoid an unwanted “surprise” in various clinical and surgical situations.
